# Impact of the Extent of Surgical Resection on Patients With Intradural Extramedullary Bronchogenic Cysts: A Retrospective Institutional Experience and Review of the Literature

**DOI:** 10.3389/fneur.2021.706742

**Published:** 2021-12-02

**Authors:** Jinghui Liu, Yuan Wang, Chen Li, Peigang Ji, Shaochun Guo, Yulong Zhai, Na Wang, Miao Lou, Meng Xu, Min Chao, Fuqiang Feng, Ming Yan, Liang Wang

**Affiliations:** ^1^Department of Neurosurgery, Tangdu Hospital, The Fourth Military Medical University, Xi'an, China; ^2^Department of Neurosurgery, The Second Hospital of Shanxi Medical University, Taiyuan, China; ^3^Institute of Orthopaedic Surgery, Xijing Hospital, The Fourth Military Medical University, Xi'an, China

**Keywords:** bronchogenic cyst, intradural extramedullary, intraspinal cyst, endodermal cyst, surgery

## Abstract

Intradural extramedullary bronchogenic cysts (IEBC) are rare congenital cystic lesions. The clinical manifestations, radiological characteristics, especially the optimal treatment regimen are not well-understood. We retrospectively analyzed a series of patients with confirmed IEBC in Tangdu hospital and reviewed the published works to gain a comprehensive understanding of IEBC. In our institution, nine consecutive patients had pathologically confirmed IEBC between 2005 and 2018. We also identified 27 patients from previous studies. The most common presentations on magnetic resonance imaging (MRI) were hypointensity on T1-weighted images (T1WI), hyperintensity on T2-weighted images(T2WI), and no improvement on T1WI contrast-enhanced with gadolinium (94.4%). All patients in our center and the patients we reviewed received surgical resection; gross total resection (GTR) and partial resection (PR) were achieved in 20 (55.6%) and 16 (44.4%) patients, respectively. The symptom remission rate of patients who underwent GTR was 100%, which was similar to those who underwent PR (93.8%) (*P* = 0.457). The recurrence rate was 12.5% in the group who underwent PR and nil after GTR (*P* = 0.202). According to our current investigation, the surgical resection degree is irrelevant to the symptom remission rate. Therefore, we suggest that total resection should not be recommended for cases with tight adhesion. For patients with PR, longer follow-up will be necessary to determine the long-term outcome.

## Introduction

Bronchogenic cysts are benign congenital malformations derived from the primitive foregut and are often classified as among the histopathological subtypes of neurenteric cysts (1). Bronchogenic cysts have been found to be attached to the sternum, pericardium, and skin and within the diaphragm (2). Intraspinal cysts are extremely uncommon; several case reports suggested that the site of predilection is the cervical or upper thoracic segments generally with an intradural extramedullary localization (3). The symptoms are associated with the location of lesions in the spinal canal. The most common symptoms are pain and sensory disturbances in the neck, back, and even the extremities (4).

To date, only a few Intradural extramedullary bronchogenic cysts (IEBC) patients have been reported. Because of its extremely low morbidity, the clinical features and optimized treatment strategy remain poorly understood. We retrospectively analyzed a series of patients with confirmed IEBC at our hospital and reviewed the published works to gain a comprehensive understanding of IEBC, including the age of onset, clinical manifestations, imaging characteristics, surgical strategies, symptom remission rate, and recurrence rate.

## Materials and Methods

All patients who had undergone surgical resection and had pathologically confirmed IEBC between 2005 and 2018 were identified *via* a review of all consecutive patients performed at our hospital. We excluded patients with a diagnosis of bronchogenic cysts that were not located in intraspinal and/or intradural intramedullary lesions. The systematic literature review was conducted in PubMed. Bronchogenic cyst, intradural bronchogenic cyst, and spinal bronchogenic cyst were the keywords for the searching. The search was restricted to English-language publications with no date limitations. Any case report that involving histopathological confirmed IEBC was included. An analysis on patients with IEBC in previous literature, together with the patients identified in our institution, was conducted to comprehensively understand the disease. The local Medical Ethics Review Committee confirmed that ethical approval was not required.

Patients who were identified in our hospital and in previous studies were investigated for the following data: sex, age, preoperative symptoms, radiological features, symptom duration, surgical findings, the extent of resection, postoperative symptoms, and long-term outcomes. The extent of resection was classified as GTR (>95%) and PR (<95%) based on operation note or postoperative magnetic resonance imaging (MRI).

Symptom remission was defined as a reduction in the symptoms before being discharged after the operation. Recurrence was defined once the lesion was increased significantly based on MRI during the follow-up. The symptom remission rate was defined as the proportion of patients who achieved remission of symptoms after operation, whereas the recurrence rate was defined as the proportion of patients with disease recurrence.

### Statistical Analysis

Statistical analysis was conducted using SPSS version 22 (IBM, Corporation). The frequencies of dichotomous variables were calculated, and numerical variables were expressed as mean and standard deviations if they were normally distributed or as median if they were not normally distributed. Symptom remission and recurrence rates were expressed as percentages and were compared using Fisher's exact test. *P* < 0.05 was defined as statistically significant.

## Results

### Patient Demographics

**Figure 1:**
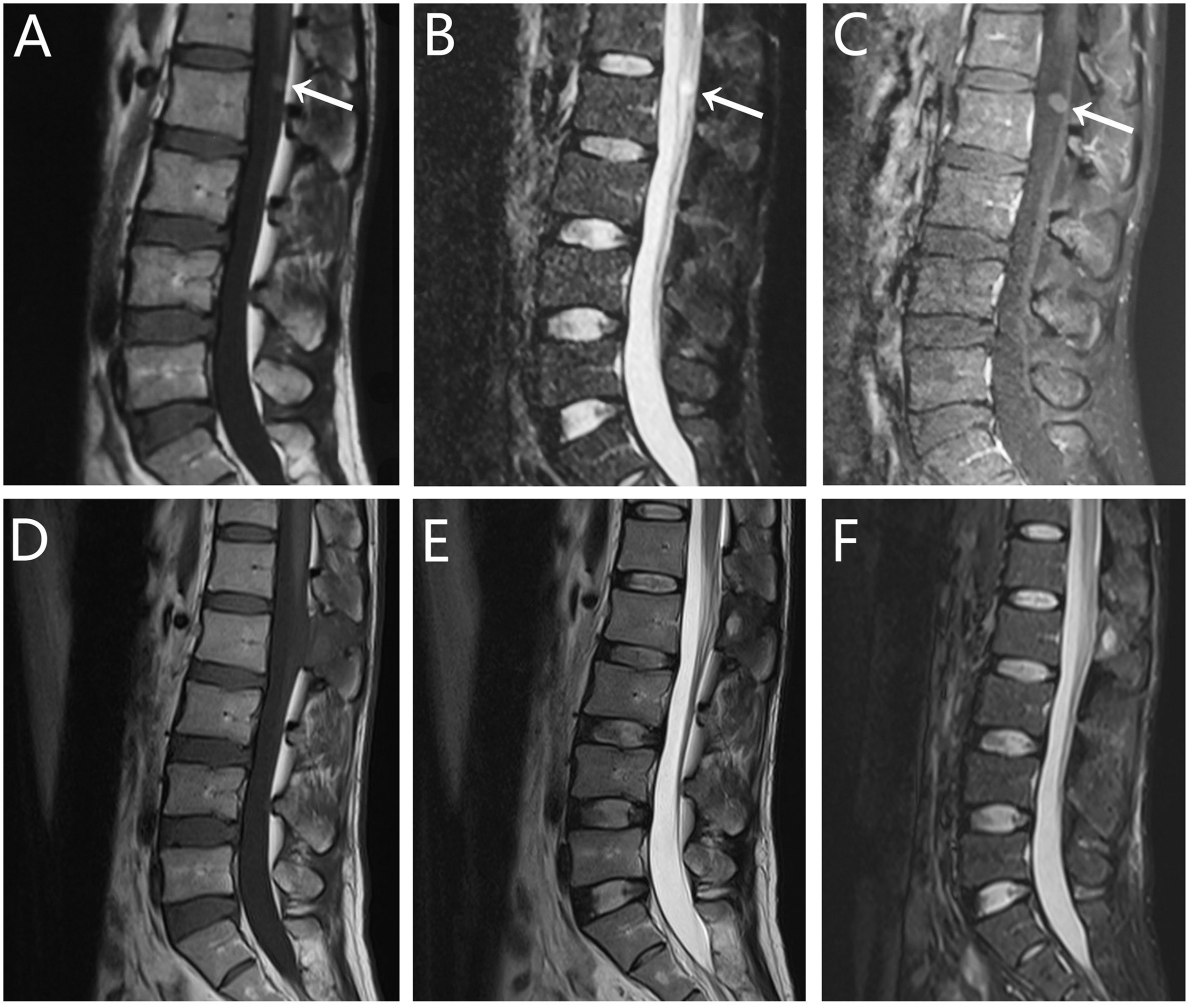
Radiographic images of case 1. **(A–C)** Pre- and **(D–F)** postoperative magnetic resonance imaging (MRI) scans revealed a cystic lesion dorsal to the L2 spinal cord. Sagittal MRI scan demonstrated isointense signal on **(A)** T1-weighted imaging (T1WI) and hyperintense signal on **(B)** T2-weighted imaging (T2WI). **(C)** T1WI with gadolinium administration showed slightly enhanced. After 11 months of follow-up, the re-examination MRI showed that the lesion was totally resected, with no evidence of recurrence.

In our institution, we totally reviewed nine patients who received surgery and confirmed IEBC from 2005 to 2018. Table 1 presents the clinical related information of these cases. The median age at diagnosis was 33 years (range, 7–55 years). The median symptom duration was 1 months (range, 0.5–48 months). All the patients were symptomatic, and the preoperative clinical symptoms included lumbodorsal pain in four patients, back pain and left lower extremities pain in one patient, weakness of lower extremities in two patients, lower extremities numbness in two patients, and back numbness in one patient. All patients underwent an MRI examination before the operation. There were total ten lesions in nine patients, with one patient bearing two lesions. Six lesions were located at the lumbar vertebra, three at the thoracic vertebra, and another one at the thoracolumbar junction. MRI indicated long T1 signal in seven patients and isointense T1 signal in two patients. All patients presented long T2 signal on MRI scan. Only one showed a slightly enhanced signal on T1WI contrast-enhanced with gadolinium (Figure 1), and the boundaries were well-demarcated. There were five patients combined with multiple congenital spinal anomalies. All patients received operative treatment; GTR was achieved in seven cases while PR in two cases. Posterior fusion with instrumentation was carried out when laminectomy was performed in three or more levels. There were no severe complications during and after surgery. Histopathological findings confirmed bronchogenic cysts in all cases in our series. All patients got relief from their preoperative symptoms after surgery. The median follow-up was 23 months, and no recurrence was reported in our cases.

**Table 1:** Clinical data and outcome of IEBC in our hospital.

**Case**	**Age**	**Symptoms**	**Location**	**Radiological findings**			**Anomaly**	**EOR**	**FU months**	**Symptom relief**	**Recur**
***N***	**Sex**			**T1WI**	**T2WI**	**T1CE**					
1	33-year M	Lumbodorsal pain for 2 weeks	Dorsal L2	ISO	Hyper	SE	No	GTR	62	Yes	No
2	21-year M	Back pain, numbness and weakness in both leg for 1 month	Dorsal T1-T7	Hypo	Hyper	No	No	PR	23	Yes	No
3	20-year M	Back pain and numbness in both leg for 1 month	Dorsal L3-L5	ISO	Hype	No	Scoliosis	GTR	62	Yes	No
4	41-year M	Right lumbodorsal pain for 3 years	Dorsal L2	Hypo	Hyper	No	SBO	GTR	12	Yes	No
5	10-year M	Scoliosis was found for 1 month	Dorsal T2-T3	Hypo	Hyper	No	Scoliosis	GTR	60	Yes	No
6	50-year M	Lumbodorsal pain, numbness in both leg for 2 months	Ventral L1	Hypo	Hyper	No	No	GTR	58	Yes	No
7	42-year F	Weakness in right leg and left lumbodorsal pain for 2 weeks	Lateral L2-L3	Hypo	Hyper	No	Scoliosis	GTR	11	Yes	No
8	55-year M	Numbness in back for 4 years	Ventral T2-T3	Hypo	Hyper	No	No	GTR	3	Yes	No
9	7-year F	Both leg pain and urinary incontinence for 4 years	Two lesions L1	Hypo	Hyper	No	LM, TSC, SBO	PR	23	Yes	No

*IEBCs, intradural extramedullary bronchogenic cysts; M, male; F, female; T1WI, T1-weighted imaging; T2-WI, T2-weighted imaging; T1CE, T1 contrast-enhanced; Hyper, hyperintense; Hypo, hypointense; ISO, isointense; SE, slightly enhanced; SBO, spina bifida occulta; LM, lumbosacral meningocele; TSC, Tethering spinal cord; EOR, extent of resection; GTR, gross total resection; PR, partial resection; FU, follow-up*.

### Comprehensive Demographics Data

**Figure 2:**
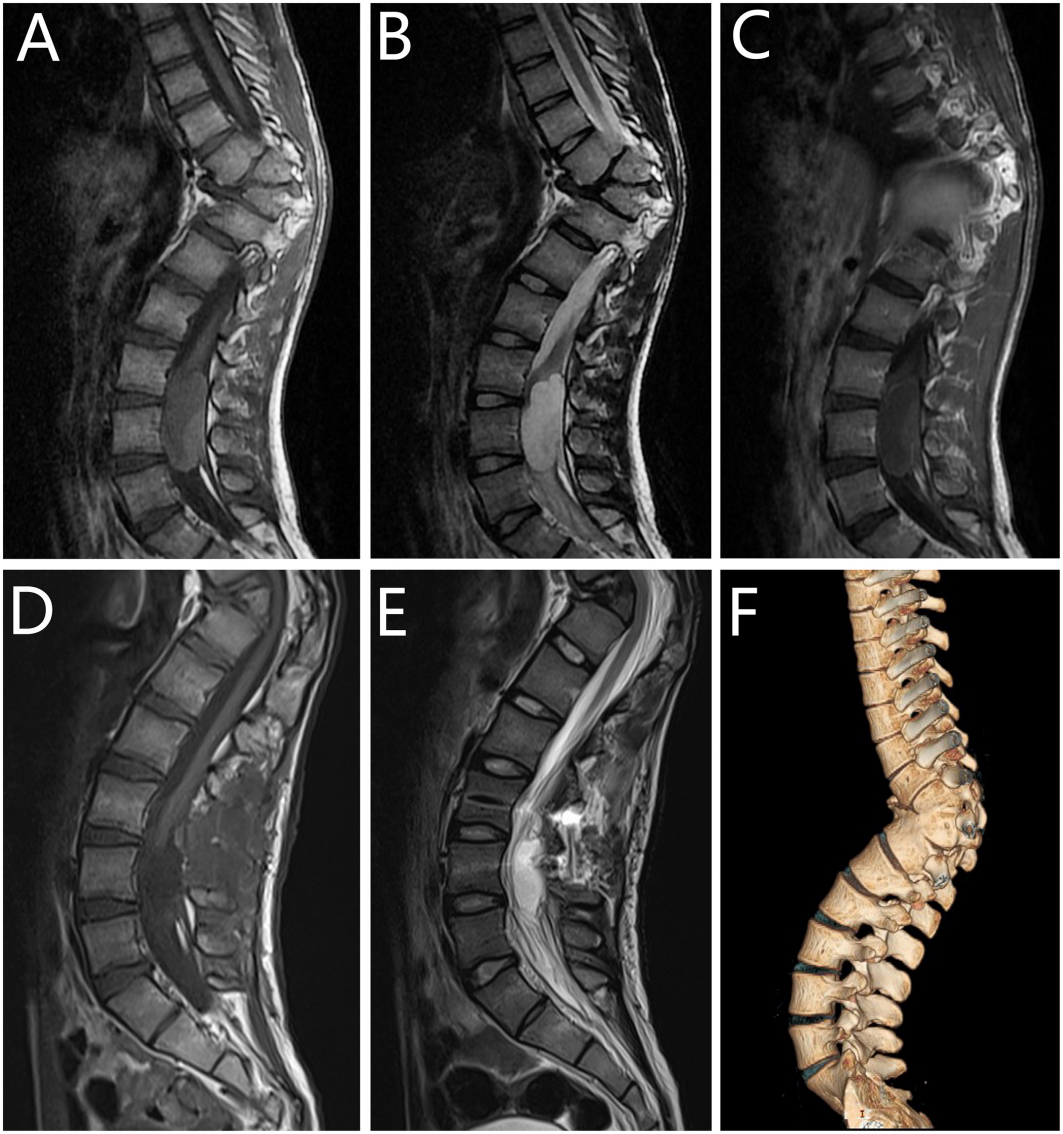
Radiographic images of case 3. **(A–C)** Pre- and **(D,E)** postoperative magnetic resonance imaging (MRI) scans revealed a cystic lesion dorsal to the L3-L5 spinal cord. Three-dimensional reconstruction **(F)** illustrated scoliosis in the thoracolumbar region. Sagittal MRI scan demonstrated isointense signal on **(A)** T1-weighted imaging (T1WI) and hyperintense signal on **(B)** T2-weighted imaging (T2WI). **(C)** T1WI with gadolinium administration showed no enhancement after administration of the intravenous contrast material on T1WI. After 6 months of follow-up, the re-examination MRI showed that the lesion was totally resected.

A total of 27 patients were identified from the literature (1, 3–19), pooled with the nine new patients described above. Table 2 shows the patient clinical baseline characteristics and radiologic findings for all 36 patients. The median age at diagnosis was 31 years (range, 0.5–66 years). With 21 males, 14 females, and 1 unreported. Patients often presented with multiple symptoms. The most common symptom presented was pain (*n* = 29), followed by paresthesia (*n* = 16), and weakness (*n* = 9). Other symptoms included urinary incontinence (*n* = 2), double upper limb tremor (*n* = 1), skin dimple in the sacral area (*n* = 1), and scoliosis (*n* = 1). The median duration of symptoms was 2 months (range 0.17–108 months) prior to presentation to the hospital. Fifteen patients had congenital spinal anomaly. The most common anomaly was scoliosis (*n* = 7) (Figure 2) and spina bifida occulta (*n* = 5), followed by spinal fusion (*n* = 3), tethered cord (*n* = 3), and spinal meningocele (*n* = 3). Four patients presented with multiple malformations.

**Table 2:** Summary all the cases of IEBCs.

**References**	**Case**	**Age, years**	**Location**	**Radiological findings**			**Anomaly**	**EOR**	**FU months**	**Symptom relief**	**Recur**
	***N***	**Sex**		**T1WI**	**T2WI**	**T1CE**					
Yamashita et al. (5)	1	14/F	C6-CT	NR	NR	NR	SBO, VF	GTR	11	Yes	No
Ho and Tiel (6)	2	21/F	C5-T3	NR	NR	NR	No	GTR	NR	NR	Nr
Wilkinson et al. (7)	3	55/F	C3-C4	NR	NR	NR	Scoliosis	PR	12	Yes	No
Baba et al. (8)	4	16/M	C1	ISO	hyper	NR	No	PR	12	Yes	No
Rao et al. (9)	5	18/M	C2-C3	Hypo	hyper	NR	No	GTR	3	Yes	No
Slowinski et al. (10)	6	43/F	C4	Hypo	hyper	NR	VF	GTR	36	Yes	No
Baumann et al. (11)	7	41/NR	T12-L1	NR	hyper	No	SBO	PR	3	No	No
Ko et al. (3)	8	28/M	L1	NR	hyper	NR	No	PR	NR	Yes	No
Chongyi et al. (12)	9	0.42/F	S2	Hypo	hyper	NR	TSC, SBO	GTR	NR	Yes	No
Arnold et al. (13)	10	20/M	T4	NR	hyper	NR	No	GTR	1	Yes	No
Solaroglu et al. (14)	11	50/F	Brainstem	ISO	hyper	No	No	GTR	3	Yes	No
Liu et al. (15)	12	55/M	T5-T6	Hypo	hyper	NR	No	PR	12	Yes	No
Zou et al. (1)	13	44/F	L3-L4	MHI	hyper	NR	TSC	GTR	6	Yes	No
Chen et al. (4)	14	24/M	L4-L5	ISO	hyper	No	LM	PR	NR	Yes	No
	15	29/M	T9-T10	Hypo	hyper	No	Scoliosis	PR	NR	Yes	No
	16	34/M	CCJ	Hypo	hyper	No	No	GTR	NR	Yes	No
Lee et al. (16)	17	44/M	T12-L1	Hypo	hyper	NR	No	GTR	1	Yes	No
Ma et al. (17)	18	23/F	C4-C7	Hypo	hyper	No	VF, Scoliosis	PR	6	Yes	No
	19	37/F	C4-C6	Hypo	hyper	No	No	PR	NR	Yes	No
	20	66/M	L1-L2	NR	NR	SE	No	PR	NR	Yes	No
Jha et al. (18)	21	43/M	C2-C4	Hypo	hyper	No	No	GTR	12	Yes	No
Weng et al. (19)	22	23/M	C3-C4	Hypo	hyper	No	No	PR	54	Yes	No
	23	15/M	L1-L2	MHI	MHI	No	No	PR	47	Yes	Yes
	24	25/F	C2-C4	Hypo	hyper	No	No	GTR	42	Yes	No
	25	41/F	C4	Hypo	hyper	No	NO	PR	81	Yes	No
	26	6/M	C2-C5	Hypo	hyper	No	Scoliosis	PR	23	Yes	Yes
	27	36/F	CCJ	Hypo	hyper	No	No	GTR	12	Yes	No
Our cases	28	33/M	L2	ISO	hyper	SE	No	GTR	62	Yes	No
	29	21/M	T1-T7	Hypo	hyper	No	No	PR	23	Yes	No
	30	30/M	L3-L5	ISO	Hype	No	Scoliosis	GTR	62	Yes	No
	31	41/M	L2	Hypo	hyper	No	SBO	GTR	12	Yes	No
	32	10/M	T2-T3	Hypo	hyper	No	Scoliosis	GTR	60	Yes	No
	33	50/M	L1	Hypo	hyper	No	No	GTR	58	Yes	No
	34	42/ F	L2-L3	Hypo	hyper	No	Scoliosis	GTR	11	Yes	No
	35	55/M	T2-T3	Hypo	hyper	No	No	GTR	3	Yes	No
	36	7/F	L1	Hypo	hyper	No	LM, TSC	PR	23	Yes	No

*IEBCs, intradural extramedullary bronchogenic cysts; M, male; F, female; CCJ, craniocervical junction; T1WI, T1-weighted imaging; T2-WI, T2-weighted imaging; T1CE, T1 contrast-enhanced; Hyper, hyperintense; Hypo, hypointense; ISO, isointense; MHI, mixed hyper and iso; SE, slightly enhanced; VF, Vertebral fusion; SBO, spina bifida occulta; LM, lumbosacral meningocele; TSC, Tethering spinal cord; EOR, extent of resection; GTR, gross total resection; PR, partial resection; FU, follow-up; NR, not reported*.

### Radiographic Evaluation

**Figure 3:**
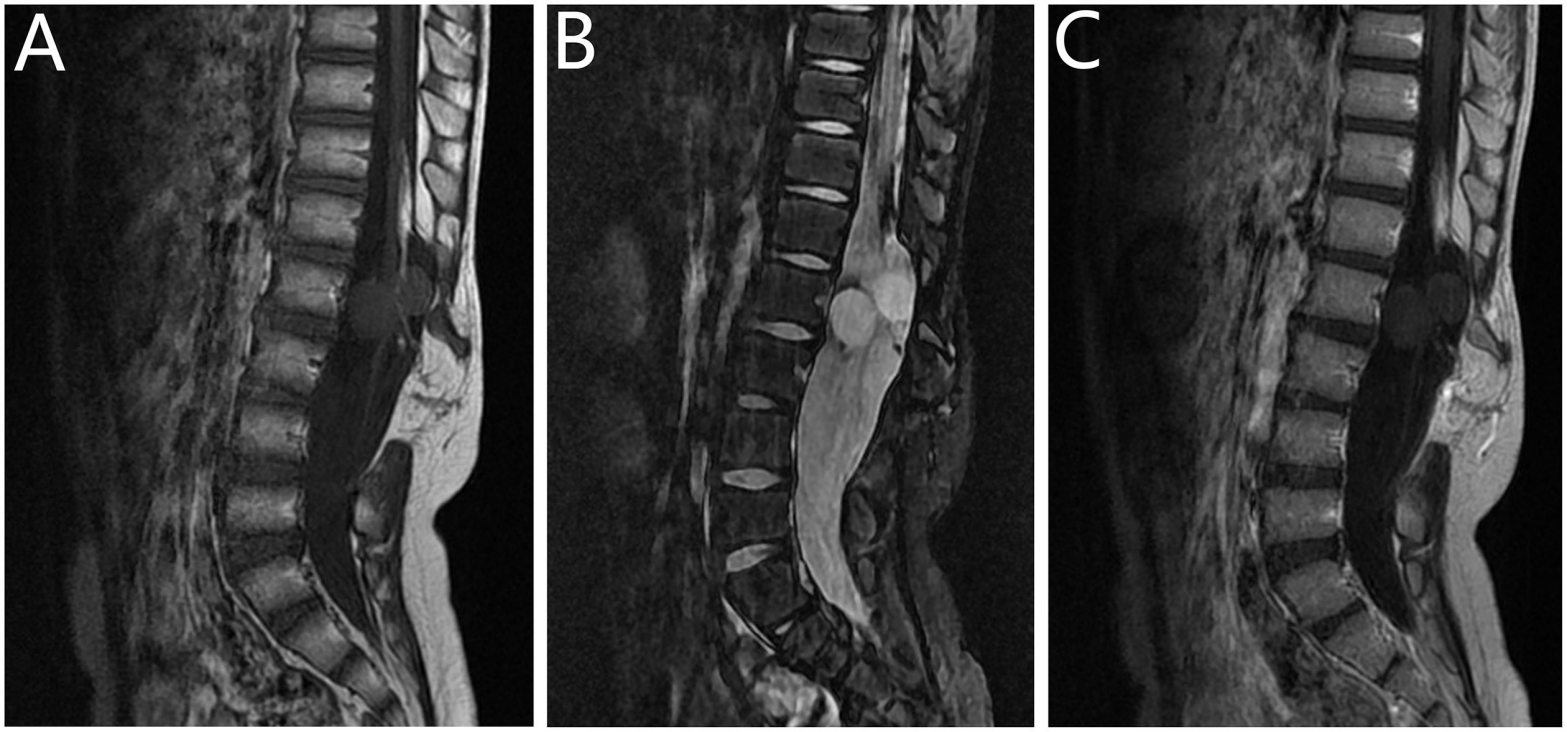
Radiographic images of case 9. Preoperative **(A–C)** magnetic resonance imaging (MRI) scans revealed two cystic lesions to the L1 spinal cord. This patient had tethered cord syndrome (with spina bifida and myelomeningocele). Sagittal MRI scan demonstrated Hypointense signal on **(A)** T1-weighted imaging (T1WI) and hyperintense signal on **(B)** T2-weighted imaging (T2WI). **(C)** T1WI with gadolinium administration showed no enhancement.

Since one of the patients had two lesions (Figure 3), there were a total of 37 lesions in 36 patients. Twenty-three lesions were hypointense on T1-weighted images (T1WI), five lesions were isointense on T1WI, while two lesions presented with a combination of hyperintense and isointense on T1WI. We could not retrieve other seven lesion images from the literature. On T2-weighted images (T2WI), except for one lesion presenting a combination of hyperintense and isointense signal, other lesions were all showed a hyperintense signal on T2WI. The radiological images of four lesions could not be retrieved from the published articles. Twenty-four patients underwent preoperative enhanced MRI, and only two (8.3%) cases showed a slightly enhanced signal. Nineteen lesions were located at the cervical and thoracic vertebra (51.4%); 12 (32.4%) cases at the lumbar vertebra; two (5.4%) cases at the thoracolumbar junction; two (5.4%) cases at the craniocervical junction; one (2.7%) cases at the brainstem; and one (2.7%) cases at the sacral vertebra. Axial MRI imaging showed that the tumor location was dorsal in 19 patients, ventral in 13 patients, and lateral in two patients, one unknown. All lesions were cystic and without obvious solid components.

### Treatment and Outcome

All but two patients underwent resection of the tumor through a posterior midline approach. The goal of surgery was to achieve the total resection of the cyst. However, this has not been attained in all patients, and only 20 (55.6%) of the patients received GTR. Because of tight adhesions to the medullary substance and cauda equina, it was difficult to conduct the total resection of IEBC. The GTR rate had no significant differences among cervical, thoracic, and lumbar vertebra. Similarly, the GTR rate did not have significant differences among dorsal and ventral (47.4 vs. 53.9%, *P* = 0.719). Follow-up was available for 11/16 (68.9%) patients who underwent PR, and the median follow-up time of these patients was 23 months (3–81 months). The median follow-up of all patients we reviewed was 12 months (1–81 months). The symptom remission rate of the patients who underwent GTR was 100%, while the remission rate for PR cases was (93.8%) (*P* = 0.457). The patients who underwent GTR had no evidence of recurrence, while two patients with PR experienced recurrence at 23 and 47 months after surgery. However, the recurrence rate between the two groups had no significant difference (*P* = 0.202).

## Discussion

The occurrence of intradural extramedullary bronchogenic cysts is extremely uncommon. Weng et al. (19) reported a series of patients with IEBC and conducted a literature review in 2018; 19 patients were identified from the literature review and pooled with six new patients they described, garnering a total of 25 patients. Since no sufficient reports were available and no study was performed to statistically analyze the clinical data of these patients, the most effective management of IEBC remains poorly understood. In the present study, for the first time, we performed a comprehensive analysis to better understand the disease characteristics and evaluate the optimum treatment of IEBC.

Congenital cervical cysts are common in pediatric populations and constitute one of the most intriguing areas of pediatric pathology. The differential diagnosis is broad, including thyroglossal duct cysts, cystic hygromas, branchial cleft cysts, bronchogenic cysts, and thymic cysts. In a 20 year retrospective review of pediatric neck cysts conducted by Hsieh et al. (20), bronchogenic cysts were found to represent <1% of the published series. In 1973, Yamashita et al. (5) reported a case of a 14 year-old girl who presented with a 2 years history of intermittent neck pain and stiffness of the left arm. In addition, this was the first report of an intradural extramedullary bronchogenic cyst. To our knowledge, there are 27 patients of IEBC reported in English literature, and after pooling with the nine new patients of our center, there were a total of 36 patients. A study has reported that 56% of all IEBC were diagnosed between 20 and 40 years old and 72.0% of them were located at the cervical and upper thoracic regions (19). Our result was in accordance with previous study. The median age of disease onset in our study was 31 years, and only 41.7% of IEBC were located in the lumbar, sacral, and lower thoracic regions.

Spinal bronchogenic cysts are benign, slow growing lesions. The clinical symptoms mainly induced by a spinal cord injury with space-occupying lesions in the spinal canal (21). According to previous studies (3), six patients presented with pain in the limbs and back, three with limb weakness, two with numbness, and only one with paresthesia. Patients commonly showed pain in the neck or back, along with radiating pain and motor and sensory disturbances in the extremities (4). These findings were consistent with our study. In our series, the most common symptom was pain (*n* = 29, 80.6%), followed by paresthesia (*n* = 16, 44.4%), and weakness (*n* = 9, 25.0%).

Due to the rarity of this disease, preoperative diagnosis remains a challenge. The basic examination of the bronchogenic cystic lesion includes computed tomography (CT) and MRI. MRI demonstrates higher resolution than CT and has been greatly emphasized. MRI characteristics usually showed homogenous and hyperintense on T2WI and hypointense on T1WI (17, 19). But enhancement was rarely after gadolinium infusion (11, 18). According to the MRI features provided in the previous studies and the patients in our hospital, we suggested that lesions with hypointense on T1WI and hyperintense on T2WI was more common.

Although MRI is superior in showing the relationship between the cystic mass and surrounding neural structures, myelograms and CT are still considered as important methods for excluding other congenital bony anomalies such as spina bifida occulta and scoliosis (22). In a study by Chen (4), including total 13 patients with IEBC, four cases (30.7%) (three with spina bifida occulta and one with scoliosis) had congenital bony anomaly. Based on our research, it can be noted that 15 (41.7%) patients had congenital spinal anomaly. The most common anomaly was scoliosis (*n* = 7) and spina bifida occulta (*n* = 5), and these present a challenge to the surgeon.

The pathogenesis of bronchogenic cysts remain unknown. A study had shown that the lesion usually grows slowly for the tight junctions between the epithelial cells (23). In addition, there was no report of malignant transformation occurring in spinal bronchogenic cysts. The most effective management strategy for intraspinal bronchogenic cysts was surgical resection. Once the cyst was removed, the patient's symptoms usually relieved. The goal of surgery was to achieve the total resection of the cyst. But for most of the patients, the goal was hard to achieve. Only 36% of the cysts reported in the literature received complete resection (24). The reasons behind the incomplete resection including blocking the surgical corridor by ventral location related to the spinal cord, and adherence of the cyst tissue to the medullary substance. In our reviewed only 20 (55.6%) received GTR. However, it was worth noting that the symptom alleviation rate in PR was similar for the rate in GTR. Thus, for patients with tight adhesion and vertebral anomalies, we suggest precluding GTR to reduce the postoperative complications. Whereas, achieving the goal of total resection is still the primary surgical treatment for IEBC. PR and cystic fenestration with no total excision of the cyst wall will increase the risk of recurrence (25). Our present study implied that the surgical resection degree was irrelevant to the recurrence rate, Considering the design of retrospective study and relatively small sample size and short follow-up time for patients with PR (median, 23 months), we still need to interpreted the result with prudence.

### Limitation

Although the findings of this study are encouraging, it had some limitations. Comparing to other prospective clinical trials, retrospective study lacks exact parallel study design, which may lead to prudent interpretation for the results. In addition, the assessment of the EOR of 27 included cases in our current study was based on previous published literature, incomplete standardization and missing detailed baseline data were inevitable for certain cases. This is considered to be a drawback of the retrospective design. Due to the rarity of the disease, the case number is relatively small and span of following up varies for years. It is possible that the symptom relief might be higher in patients with GTR. However, our current data did not support this deduction. Therefore, we suggest that future larger case study with longer follow-up is recommended for better understanding the prognostic factors for the disease.

## Conclusion

IEBC is extremely rare. Together with our nine cases series, there were only total 36 patients could be analyzed. The most observed locations of IEBC were cervical and thoracic vertebra, and the primary symptom was pain. To our best knowledge, this retrospective study represented the largest number of IEBC cases reported to date in the literature, and we analyzed the relationship between the surgical resection and patients' prognostic benefit, which showed profound clinical implication. Surgical resection was the most effective treatment for IEBC. According to our findings, we recommend that GTR should not be suggested for cases with tight adhesion. Larger sample size and longer follow-up was advised for future studies to better understanding the disease.

## Data Availability Statement

The original contributions presented in the study are included in the article/Supplementary Material, further inquiries can be directed to the corresponding author/s.

## Author Contributions

JL performed most of the data analyses and drafted the manuscript. YW performed most of the clinical analyses (imaging data) together with CL and contributed to the writing of the manuscript. PJ contributed to the data analyses together with SG and YZ. NW performed most of the clinical follow-up with MX and MC. LW performed the clinical analyses and designed the study together with JL and FF. MY performed supervised analyses and made crucial revisions to the manuscript. All authors contributed to the article and approved the submitted version.

## Funding

This work was funded by the National Natural Science Foundation of China (81772661).

## Conflict of Interest

The authors declare that the research was conducted in the absence of any commercial or financial relationships that could be construed as a potential conflict of interest.

## Publisher's Note

All claims expressed in this article are solely those of the authors and do not necessarily represent those of their affiliated organizations, or those of the publisher, the editors and the reviewers. Any product that may be evaluated in this article, or claim that may be made by its manufacturer, is not guaranteed or endorsed by the publisher.
