# Supplementary material for: Impact of the Extent of Surgical Resection on Patients With Intradural Extramedullary Bronchogenic Cysts: A Retrospective Institutional Experience and Review of the Literature
Source: Front Neurol. 2021 Dec 2;12:706742. doi: 10.3389/fneur.2021.706742 (PMC8674416; doi:10.3389/fneur.2021.706742)
Supplement: Supplementary file 1 [file Table_1.DOCX]

**Supplementary table 1: Clinical Data, Surgical Management, and Outcomes of IEBCs**

| study | Case | Preoperative Symptoms | Interval of Symptoms | | EOR | Surgical complications | Recur | Treatment for relapse |
| --- | --- | --- | --- | --- | --- | --- | --- | --- |
|  | n |  | months | |  |  |  |  |
| 1973^5^ | 1 | Intermittent neck pain, pain and stiffness of the left arm | 48 | | GTR | No | No | — |
| 1989^6^ | 2 | Tingling, numbness and diminishing sensation in the right arm | 1.5 | | GTR | NR | NR | — |
| 1992^7^ | 3 | Pain and paranesthesia in right arm | 0.5 | | PR | No | No | — |
| 1995^8^ | 4 | Pain in the posterior upper neck region | 12 | | PR | No | No | — |
| 1999^9^ | 5 | Radiating pain and progressive weakness of the right upper limb | 1.5 | | GTR | No | No | — |
| 2004^10^ | 6 | Neck pain and progressive weakness of lower limbs | NR | | GTR | No | No | — |
| 2005^11^ | 7 | Continuous, sharp, and therapy-refractory pain in the left leg | NR | | PR | Yes^🞼^ | No | — |
| 2008^3^ | 8 | Chronic lumbago and progressive weakness and numbness in both lower limbs | 0.5 | | PR | No | No | — |
| 2008^12^ | 9 | Skin dimple in the sacral area | NR | | GTR | No | No | — |
| 2009^13^ | 10 | Increasing back pain and lower-extremity weakness | 6 | | GTR | No | No | — |
| 2013^14^ | 11 | Intermittent occipital headaches, neck pain, syncope attacks and sensory disturbances in her extremities | 6 | | GTR | No | No | — |
| 2015^15^ | 12 | Progressive weakness and numbness in both lower limbs | 0.17 | | PR | No | No | — |
| 2015^1^ | 13 | Low back pain and leg weakness as well as sphincter disturbance | 108 | | GTR | No | No | — |
| 2015^4^ | 14 | Progressive back pain | 1 | | PR | No | No | — |
|  | 15 | Back pain and numbness in the lower extremities | 1 | | PR | No | No | — |
|  | 16 | Neck pain and left leg numbness | 6 | | GTR | No | No | — |
| 2017^16^ | 17 | Weakness in left lower extremity, radiating pain in the left hip and posterior thigh with low back pain | 21 | | GTR | No | No | — |
| 2017^17^ | 18 | Pain in the right upper limb | 1 | | PR | No | No | — |
|  | 19 | Neck pain and numbness in both upper limbs | 0.5 | | PR | No | No | — |
|  | 20 | Lower back pain | 0.3 | | PR | No | No | — |
| 2018^18^ | 21 | Gradually progressive neck pain radiating to the right shoulder | 4 | | GTR | No | No | — |
| 2018^19^ | 22 | Neckache | 4 | | PR | No | No | — |
|  | 23 | Lumbodorsal pain | 10 | | PR | No | Yes | Surgery |
|  | 24 | Neckache and double upper-limb  numbness | 2 | | GTR | No | No | — |
|  | 25 | Neck shoulder numbness | 12 | | PR | No | No | — |
|  | 26 | Double upper limb tremor | 36 | | PR | No | Yes | Observation |
|  | 27 | Occipitocervical pain | 1 | | GTR | No | No | — |
| Our cases | 28 | Lumbodorsal pain | 0.5 | | GTR | No | No | — |
|  | 29 | Back pain, numbness and weakness in both legs | 1 | | PR | No | No | — |
|  | 30 | Back pain and numbness in both legs | 1 | | GTR | No | No | — |
|  | 31 | Right lumbodorsal pain | 36 | | GTR | No | No | — |
|  | 32 | Scoliosis was found | 1 | | GTR | No | No | — |
|  | 33 | Lumbodorsal pain, numbness in both legs | 2 | | GTR | No | No | — |
|  | 34 | Weakness in right leg and left lumbodorsal pain | 0.5 | | GTR | No | No | — |
|  | 35 | Numbness in back | 48 | | GTR | No | No | — |
|  | 36 | Both legs pain and urinary incontinence | 48 | PR | | No | No | — |

IEBCs, intradural extramedullary bronchogenic cysts; EOR, extent of resection; GTR, gross total resection; PR, partial resection. NR, not Reported.

^🞼^ The patient experienced a recurrent lumbovertebral pain in both legs 3 months after surgery.
